# Parallel signatures of cognitive maturation in primate antisaccade performance and prefrontal activity

**DOI:** 10.1016/j.isci.2024.110488

**Published:** 2024-07-11

**Authors:** Junda Zhu, Xin Maizie Zhou, Christos Constantinidis, Emilio Salinas, Terrence R. Stanford

**Affiliations:** 1 Program in Neuroscience, Vanderbilt University, Nashville, TN 37235, USA; 2 Department of Biomedical Engineering, Vanderbilt University, Nashville, TN 37235, USA; 3 Department of Ophthalmology and Visual Sciences, Vanderbilt University Medical Center, Nashville, TN 37235, USA; 4 Department of Translational Neuroscience, Wake Forest University School of Medicine, Winston-Salem, NC 27157, USA

**Keywords:** Biological sciences, Developmental neuroscience, Neuroscience

## Abstract

The ability to suppress inappropriate actions and respond rapidly to appropriate ones matures late in life, after puberty. We investigated the development of this capability in monkeys trained to look away from a lone, bright stimulus (antisaccade task). We evaluated behavioral performance and recorded neural activity in the prefrontal cortex both before and after the transition from puberty to adulthood. Compared to when young, adult monkeys processed the stimulus more rapidly, resisted more effectively the involuntary urge to look at it, and adhered to the task rule more consistently. The spatially selective visuomotor neurons in the prefrontal cortex provided neural correlates of these behavioral changes indicative of a faster transition from stimulus-driven (exogenous) to goal-driven (endogenous) control within the time course of each trial. The results reveal parallel signatures of cognitive maturation in behavior and prefrontal activity that are consistent with improvements in attentional allocation after adolescence.

## Highlights

• Antisaccades typify a conflict between voluntary and involuntary forms of attention
• Both monkey performance and prefrontal activity changed markedly after adolescence
• Performance improved in parallel with the prefrontal representation of the task goal
• Effective attentional allocation is likely key for achieving robust response control

## Graphical abstract

**Figure:**
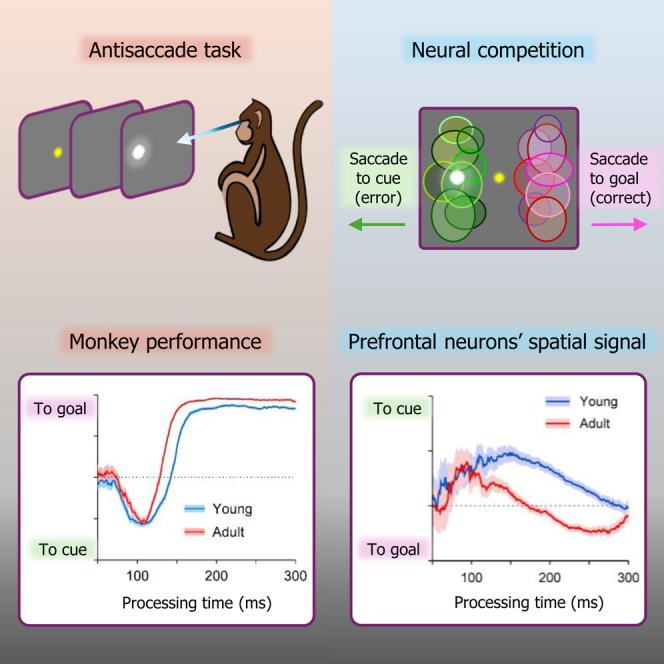

## Introduction

The antisaccade task^1^^,^^2^ requires subjects to resist the strong tendency to look toward a suddenly appearing visual stimulus and instead look to a diametrically opposed location. Because of these unique requirements, the antisaccade task has been used as an assay of “response inhibition”, a psychological construct purported to track with the maturation of behavioral control.^3^ Human antisaccade performance improves throughout childhood and adolescence^4^^,^^5^^,^^6^^,^^7^^,^^8^^,^^9^ and analogous changes have been observed in developing monkeys.^10^^,^^11^ Error rates and reaction times (RTs) decrease into adulthood,^10^^,^^11^^,^^12^ with human imaging^12^^,^^13^^,^^14^^,^^15^^,^^16^^,^^17^ and monkey neurophysiological^10^^,^^11^ studies suggesting that post-pubertal changes in the dorsolateral prefrontal cortex (dlPFC) and associated networks could underlie these gains.

The concept of inhibitory control and the role of frontal and allied networks in achieving it has been applied to a range of behaviors,^18^^,^^19^ including the antisaccade task and the countermanding or stop-signal task, which requires withholding an impending motor response.^20^ However, in both cases, the demonstration of a neural inhibitory process that is directly and specifically responsible for the psychophysically defined stopped process—i.e., a withheld movement toward a salient stimulus—has been elusive. In the case of antisaccades, the straightforward hypothesis that dlPFC provides a discrete inhibitory control signal to downstream oculomotor structures^2^^,^^21^^,^^22^^,^^23^^,^^24^ has been rejected empirically based on electrophysiological findings in monkeys.^25^^,^^26^^,^^27^ Likewise, evidence for a neural inhibitory substrate that is specifically causal to countermanding task performance has yet to be identified.^28^^,^^29^ Rather, the existence of such an inhibitory process has been inferred from the timing of prefrontal visuomotor (VM) activity,^30^^,^^31^^,^^32^ the very same activity that has been identified as a neural substrate for visuospatial attention and/or saccadic motor planning in many other experiments.^33^^,^^34^^,^^35^^,^^36^^,^^37^^,^^38^^,^^39^ Studies in human subjects, which aim to relate non-invasive measures of neural activity (e.g., EEG) to the same psychophysical markers of putative inhibitory control, are similarly challenged in their efforts, with recent reviews suggesting that variance in these measures may be better explained by functional capacities that are not inherently inhibitory.^40^^,^^41^

Rather than inhibitory control, we suggest that behavioral and neural correlates of antisaccade performance and their developmental trajectories are more easily understood in terms of spatial attentional dynamics. It has been argued that, in general, motor inhibition and attentional orienting are inseparable,^42^ but our specific reasoning is 2-fold. First, the antisaccade task engages stimulus-driven (exogenous) and goal-driven (endogenous) attentional mechanisms in intuitively obvious ways; initially, attention is drawn exogenously to the highly salient cue and, subsequently, the process of planning the antisaccade must entail the endogenous allocation of attention to its endpoint.^43^^,^^44^ Second, in contrast to an explanatory framework based on a theoretical inhibitory process, one based on attention maps readily onto well characterized neural substrates—namely, the post-stimulus (exogenous) and pre-saccadic (endogenous) activations of prefrontal VM neurons.^33^^,^^37^^,^^39^^,^^45^^,^^46^^,^^47^^,^^48^^,^^49^ Importantly, the attentional framework makes specific predictions that can be tested empricially (see the following section).

**Figure 1:**
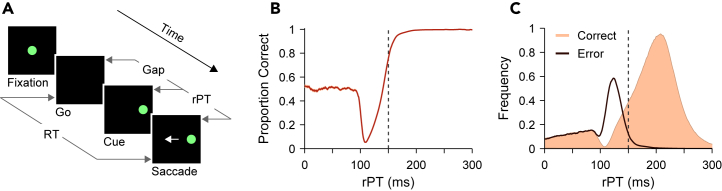
Exogenous and endogenous contributions to antisaccade performance are clearly dissociable based on processing time (A) Sequence of events in the compelled antisaccade task, an urgent version of the classic antisaccade paradigm. The go signal (Go, fixation point offset) is given first and the cue stimulus (Cue, appearing randomly at ± 10°) follows after an unpredictable delay period between them (Gap, 0–350 ms). The time window for responding is limited (RT < 450 ms), so the goal direction must be determined while motor plans advance. Performance in each trial is dictated by the raw processing time (rPT), which is the interval during which the cue can be viewed and analyzed before commitment to a choice. (B) Fraction of correct choices as a function of rPT, or tachometric curve. Performance is initially dominated by guesses (accuracy near chance), then by captured saccades (accuracy below chance), and then by informed choices (accuracy above chance). The shown curve is based on simulated data from a model that replicated in detail the performance of human participants.^51^ (C) Distributions of rPT values for correct (orange) and error (black) trials from the same simulations as in B. Dotted lines in B and C mark the rPT at which 75% correct is achieved.

With this in mind, the current study presents new analyses of previously collected behavioral and neurophysiological data.^10^^,^^11^ This work is directly inspired by recent human studies,^50^^,^^51^ so it is useful to briefly review their main result. In the human antisaccade experiments, the full scope of the exogenous/endogenous temporal dynamic was revealed by introducing a gap between the offset of the fixation point and the onset of the cue (Figure 1A, Gap), and by imposing a response deadline. Together, these elements created ideal conditions for generating a “tachometric curve” (Figure 1B), a psychometric function describing how performance changes as a function of the amount of time available to view and process the cue prior to saccade commitment (Figure 1A, raw processing time [rPT]). This processing time interval ranges from zero (when cue and saccade onsets are simultaneous) to the full RT interval (when cue and go onsets are simultaneous), and is the key determinant of performance. Early on (rPT ≈110 ms), the exogenous pull toward the salient cue is evident as a steep decline in the percentage of correct trials (Figure 1B) and a concomitant abrupt increase in error rate (Figure 1C, black trace), which correspond to the involuntary capture of saccades. Later (rPT ≈150 ms), the likelihood of exogenous capture declines and increasingly cedes to correct choices informed by the endogenously defined antisaccade rule (Figure 1C, orange). So, for a given trial, whether voluntary or involuntary attention prevails, and thus whether the choice is correct or incorrect, depends fundamentally and acutely on the processing time.

With this perspective, the current study reexamines behavioral and neurophysiological data collected from monkeys performing a gap variant of the antisaccade task at pre-adolescent and adult stages of development.^10^^,^^11^ We conceptualize monkey antisaccade performance as a rapidly advancing competition between exogenous and endogenous signaling in an effort to understand the maturation of behavioral control and its neural basis. Our objectives are, first, to determine if performance gains previously observed in developing monkeys^10^ reflect changes in the exogenous/endogenous temporal dynamic; and second, to determine if any such changing temporal signature exists in the spatial signaling of single prefrontal neurons that represent the salient cue and the antisaccade goal.

## Results

**Figure 2:**
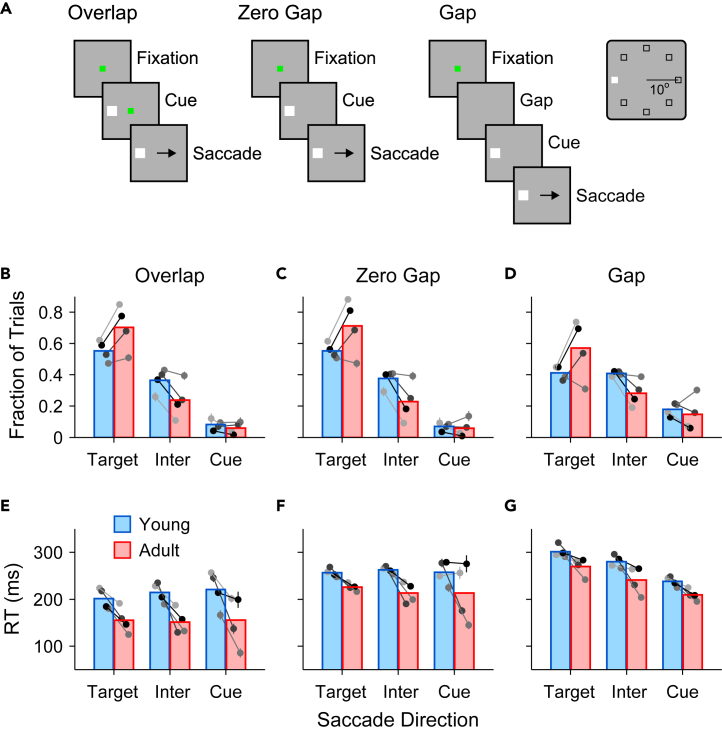
Basic manifestations of maturation on antisaccade performance (A) Sequence of events in the antisaccade task. Left, overlap variant. The cue and fixation point overlap for 100 ms before they both turn off and signal the requirement for a saccade away from the cue. Middle, zero-gap variant. The fixation point turns off simultaneously with the cue onset. Right, 100 ms gap variant. The fixation point turns off, and after a 100 ms gap, the cue appears. Inset depicts possible locations of the target on the screen. (B–D) Saccadic responses in young (blue bars) and adult monkeys (red bars). Eye movements were classified as directed to the target (Target; saccade within ±45° of target direction), directed to the cue (Cue; saccade within ±45° of cue direction), or intermediate (Inter; saccade more than ±45° away from both target and cue directions). Bars show mean proportions of responses of each type in the overlap (B), zero-gap (C), and 100 ms gap (D) conditions. Points show data from individual monkeys, with error bars indicating ±1 SEM (these are almost always smaller than the symbols). (E–G) Mean reaction time (RT) from both correct and incorrect trials for the overlap (E), zero gap (F), and 100 ms gap (G) conditions. Bars correspond to mean values; points correspond to data from individual monkeys, with error bars showing ±1 SEM.

Four male macaque monkeys (*Macaca mulatta*) performed three variants of the antisaccade task^10^ (Methods) with the key difference between them being when the 100 ms visual cue was presented relative to when the fixation point was extinguished (Figure 2A). As developed in the following text, it is the 100 ms gap condition that is critical to the analysis of behavior with respect to rPT. Because the fixation requirement is released before the salient cue is presented, the cue occurs at a time when the saccadic system is primed for movement. This timing regime increases the likelihood of producing short intervals between cue and saccade onset—short rPTs that, in human subjects, favor exogenous capture by the visual cue^50^^,^^51^ (Figure 1).

As discussed, the tachometric curve resolves the rPT-dependent influences of exogenous and endogenous factors to performance, and thus opens the possibility of determining how their dynamics change with development. If, as believed, the maturation of response control is the result of an improved ability to resist exogenous capture, this would be uniquely evident when accuracy is plotted as a continuous function of rPT (i.e., the tachometric curve; Figure 1B); specifically, the prediction is a weaker dip in performance at early rPTs (manifest as a change in steepness, depth, or duration) and/or a more robust rise of the endogenously driven recovery at later rPTs (manifest as a change in onset or slope). Importantly, such a behavioral effect predicts a correspondingly dynamic rPT-dependent neural correlate in the transition from visual cue to antisaccade goal representation, a transition expected to be more robust (e.g., faster, stronger) in adult-stage animals. To examine these predictions, behavioral data were analyzed from two stages of development: after the onset of puberty (young stage) and after morphometric and other growth measures had plateaued (adult stage), as previously described^10^^,^^11^ (Methods).

### Behavioral correlates of development

**Figure 3:**
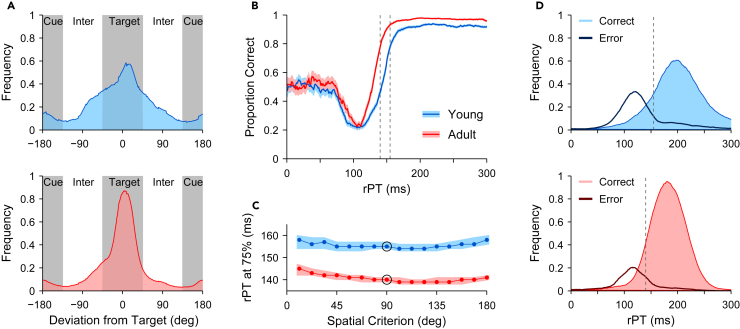
Antisaccade performance as a function of processing time Data in all panels are pooled over the four monkeys. (A) Normalized distributions (running histograms; bin size = 18°) of saccade directions in the young (top, blue) and adult (bottom, red) stages. Shades indicate the 90° spatial criterion used to classify each saccade as either directed to the target (Target, correct), to the cue (Cue, incorrect), or to neither (Inter, intermediate). Densities for young and adult, respectively, are based on 27,670 and 27,910 trials with processing times in the 0–300 range. (B) Tachometric curves showing the proportion of correct responses at each rPT bin (bin width = 20 ms). Correct and incorrect trials were assigned using the 90° spatial criterion indicated in A. Each curve combines data from all gap conditions either in the young (blue; 16,962 correct plus incorrect trials) or adult (red; 21,606 correct plus incorrect trials) stage. Vertical lines indicate the processing times at which performance reaches 75% correct, at 140 and 155 ms. Shaded ribbons indicate standard errors based on binomial statistics. (C) The processing time at which performance reaches 75% correct (y axis) is largely insensitive to the spatial criterion used to define correct and error trials (x axis). Highlighted data points for the young (blue) and adult (red) samples correspond to the values in B. Shaded ribbons indicate 95% confidence intervals (CIs) from bootstrap. (D) Normalized rPT distributions for correct (light colors) and error (dark lines) responses scored with the same spatial criterion as in B. Data are from the 100 ms gap condition in the young (top, blue) and adult (bottom, red) samples. Dotted lines mark the same processing times as in B.

As we previously reported,^10^ significant age-related increases in performance were apparent for all three antisaccade task variants. These behavioral findings, reproduced here for reference (Figures 2B–2D; red versus blue bars), show that adult-stage animals directed a larger proportion of saccades to the antisaccade target (Target) compared to either the salient visual cue (Cue) or to an in intermediate (Inter) location. Importantly, corresponding mean RT values (Figures 2E–2G) show that monkeys not only became more accurate, but also responded sooner as adults than as juveniles. This conjunction of greater speed and accuracy for all gap conditions indicates that performance increases were not simply the result of an improved ability to withhold a saccade to better exploit the speed-accuracy tradeoff. In the current study, we found that two dissociable components contributed to this overall improvement in performance in adults. One is an increase in saccadic precision. For all gap conditions, saccade direction (Figure 3A) was less tightly distributed around the target (Target, around 0°) and cue (Cue, around ±180°) locations for young-stage (Figure 3A, top) than for adult-stage (Figure 3A, bottom) animals. Consequently, for young animals, relatively more saccades fell outside of the 90° criterion windows (40% for the young; 23% for the adult) leading to classification as intermediate (Inter, around ±90°). To fully understand this result, it is important to note that monkeys were trained to expect the cue to appear randomly in cardinal (up, down, left, and right) and non-cardinal directions. Thus, “Intermediate” errors likely reflected an uninformed (anticipatory) motor plan, which could be in any direction, modulated to varying degrees by contributions either toward the cue location or the goal location.

The other component underlying better adult performance is an increase in perceptual processing efficiency. The 100 ms gap condition (Figures 2D and 2G) is unique because early offset of the fixation point grants permission to move *before* presentation of the visual cue that informs the subject where to look. Under such circumstances, advanced motor response preparation frequently leads to saccades initiated shortly before or after the cue onset, i.e., to short rPTs that are particularly revealing of any behavioral dependencies of processing speed.^51^^,^^52^ Reconsideration of antisaccade performance with respect to rPT (Figure 3B) provides a more detailed view of the factors that drive performance and how these differ as functions of age. For both juvenile and adult-stage monkeys, accuracy varied markedly as a function of rPT (Figure 3B), recapitulating in form the tachometric curves observed for adult human subjects performing an urgent antisaccade task^50^^,^^51^ (Figure 1B). That is, tachometric curves were distinctively non-monotonic, with pronounced early declines in accuracy followed by rapid recoveries toward performance asymptotes.

The tachometric curves for the young and adult-stage monkeys took the same basic form, indicating that the interaction between early exogenous and later endogenous processes is qualitatively similar across different ages. However, detailed comparison across stages demonstrated significant differences in the relative timing of the exogenous and endogenous expressions that are indicative of more efficient perceptual processing in adults. Specifically, although adult-stage animals were qualitatively similar to juveniles in their propensity to be drawn to the salient cue at very short rPTs, their capture effect was more transient, suggesting higher resilience to the exogenous pull and a speeded ability to translate cue information into an appropriately opposed motor plan. This is apparent both as a less persistent drop in accuracy and a faster rise toward asymptotic performance for the adult-stage tachometric curves (Figure 3B). Taking 75% correct as a performance benchmark (dashed vertical lines), adult-stage animals needed 16 fewer milliseconds of cue viewing time for performance to rise from a minimum to this benchmark. Specifically, young-stage animals required 50 ms of rPT for performance to rise from a minimum at rPT = 105 ms ([97, 112] ms 95% confidence interval [CI]) to 75% correct at rPT = 155 ms ([154, 157] ms 95% CI), whereas adult-stage animals required 34 ms for performance to rise from a minimum at rPT = 106 ms ([101, 110] ms 95% CI) to 75% correct at rPT = 140 ms ([139, 141] ms 95% CI), a 32% reduction in the amount of processing time needed to achieve the same improvement in accuracy. Importantly, this difference in rPT dependence for young- and adult-stage animals was not sensitive to changing the spatial criterion used to classify saccades as correct or incorrect (Figure 3C), indicating that age-related changes in processing speed were independent of those relating to saccadic precision.

As is true for human subjects^50^^,^^51^ (Figure 1B), the accuracy of monkey subjects varies widely over a very narrow range of rPT, with performance stabilizing by ∼150 ms of cue viewing time. Notably, an adult-stage performance gain is also evident in the stable asymptotic performance level, for which rPT is not a limiting factor (Figure 3B). This difference, which persists well beyond the rPT range for exogenous capture, suggests that, as adults, monkeys are less prone to lapses in performance and more consistent in their ability to apply the antisaccade rule. This mechanism likely explains the adult-stage performance gains observed for the overlap and zero-gap task variants, for which the majority (95%) of cue viewing times were long (>150 ms). Importantly, this adult-stage improvement was not due to an increase in spatial acuity and/or saccadic precision, as, once again, this difference was relatively impervious to changes in the spatial criterion used to differentiate correct from incorrect responses (on average, the difference in asymptotic performance between adult and young samples was 0.051 across 16 tested spatial criteria between 180° and 11.25°, range = [0.041, 0.075], *p* < 10^−5^ in all cases).

Greater resistance to exogenous capture, more rapid transition to endogenous control, and fewer lapses for adult-stage animals were also evident when corresponding rPT distributions were parsed to create separate plots for error and correct trials (Figure 3D). For this analysis, we considered trials from the gap condition only. For errors (Figure 3D, dark lines), the early rPT mode for young-stage (Figure 3D, top; 121 ms in [117, 122] ms 95% CI) and adult-stage (Figure 3D, bottom; 115 ms in [113, 120] ms 95% CI) animals is quite comparable (the difference is 6 ms in [-1, 8] ms 95% CI); however, these exogenously driven errors represent a much greater fraction of the total trials in young-stage animals (21%) than in adults (12%). Likewise, in young-stage animals, a pronounced tail in the distribution for errors corresponds to the long-rPT performance lapses that limit asymptotic performance at this early stage of development. In turn, the modal rPT value for correct trials (Figure 3D, filled histograms), in large part a reflection of the time necessary to exert endogenous control, occurs 18 ms (in [10, 23] ms 95% CI) earlier for adult-stage (Figure 3D, bottom; 180 ms in [178, 183] ms 95% CI) than for young-stage (Figure 3D, top; 198 ms in [191, 202] ms 95% CI) animals. Again, results were very similar with alternative spatial criteria.

**Figure 4:**
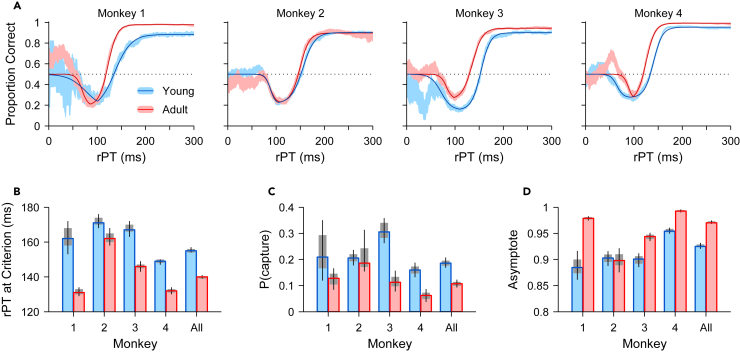
Antisaccade performance for individual subjects (A) Tachometric curves showing the proportion of correct responses at each rPT bin (bin width = 40 ms). Each curve combines trials from all gap conditions either in the young (blue) or adult (red) stage. For each curve, the light shaded ribbon denotes the mean proportion correct ±1 SE from the experimental data, and the dark trace is a continuous function fitted to those data (Methods). (B–D) Three quantities derived from the tachometric curve and used to characterize antisaccade performance for each monkey and for their combined data (All). The rPT at criterion (B) is the processing time at which performance reaches 75% correct. The probability that a saccade is captured by the cue (C) is based on how much the tachometric curve dips below chance. And the asymptote (D) is the performance level attained at long rPTs. For all quantities, bars show values for the young (blue) and adult (red) stages, and gray shades and error bars indicate 68% and 95% CIs, respectively, obtained by bootstrapping (Methods).

The development-related changes evident in the aggregate data (Figure 3B) were qualitatively consistent across each of the four individual monkeys (Figure 4A). Improved performance was evident for all four subjects as leftward shifts in the adult-stage tachometric curves (red traces) relative to those for the young-stage (blue traces). Quantified as the rPT at which performance reached 75% correct (Figure 4B), the shift toward better performance at shorter rPTs was significant for all 4 subjects, and for two subjects (monkeys 3, 4) was also accompanied by a small rightward shift of the initial downward turn in performance. These changes reflect an overall decrease in the likelihood of oculomotor capture by the cue in the adult stage, a significant effect in 3 of the 4 subjects (Figure 4C). Likewise, 3 of the 4 monkey subjects demonstrated improved asymptotic performance as adults (Figure 4D), the rPT-independent behavioral improvement suggestive of better adherence to task rules.

### Neural correlates of development

The antisaccade task specifies a motor goal that is spatially incongruent with the sensory stimulus that informs it. This defining feature of the task creates a representational conflict between exogenous mechanisms that detect and locate salient external events and endogenous mechanisms that direct attention and allied motor acts to task-relevant goals. As shown by the tachometric curve^50^^,^^51^ (Figures 1A, 3B, and 4A), success on an antisaccade task entails rapid resolution of this conflict in favor of the endogenous goal, a process that appears to become more efficient with development (Figures 3 and 4, red vs. blue data). As is typically true for brain regions implicated in visuomotor control, dlPFC and the frontal eye field (FEF) comprise a high percentage of neurons that respond in association with the sensory event and the subsequent saccade. Thus, as putative contributors to both early cue and later goal representations, we sought to determine if a counterpart of the development-related changes in antisaccade performance is evident in the timing and vigor with which the prefrontal activity transitions between these conflicting representations as an antisaccade trial unfolds.

**Figure 5:**
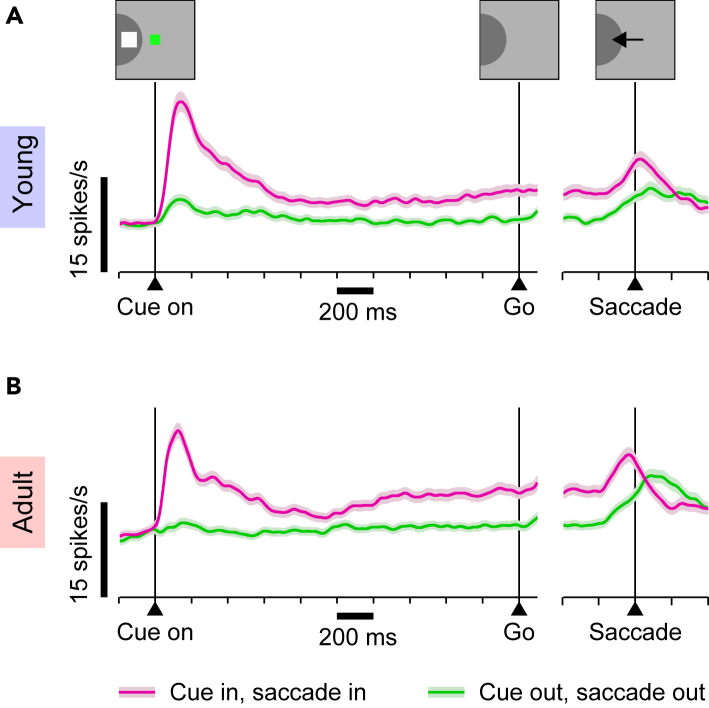
Recordings during the oculomotor delayed response (ODR) task reveal distinct cue- and saccade-related responses in PFC Results are from VM neurons, which had both visual and presaccadic activity. Traces show mean firing rates (±1 SE across neurons) as functions of time for correct saccades into the RF (saccade in, cue in; magenta) or away from the RF (saccade out, cue out; green). (A) Activity from 256 VM neurons recorded from young monkeys during performance of the ODR task. (B) As in A, but from 338 VM neurons recorded from adult monkeys.

We examined the dependence of both cue and goal representation on rPT for representative samples of young- and adult-stage prefrontal neurons. To mitigate the influence of sample bias for cell type (i.e., visual, motor, VM; see STAR Methods), for the purpose of this analysis, the samples for comparison consisted entirely of VM neurons, i.e., units classified as having spatially selective responses both after the presentation of visual stimuli and before saccades to those stimuli during performance of a standard oculomotor delayed response (ODR) task (Methods). The average activity profiles of these sample populations (Figure 5) are typical for neurons recorded in dlPFC and FEF. For stimuli in the response field (RF; Figure 5, magenta traces), neurons show a transient stimulus-contingent (cue on) response, sustained activity during the delay period, and a transient perisaccadic increase in activation. Conversely, for a stimulus/goal diametrically opposed to the RF (Figure 5, green traces), activity is greatly diminished, with increases predominantly post-saccadic.

The delay period of the ODR task temporally dissociates activity bursts contingent on the cue and saccade in a way that allows for independent assessment of their strength and timing with respect to these task events. For both young- and adult-stage animals, presentation of the cue in the RF evokes a short-latency transient response that peaks at approximately 100–150 ms and which decays to approximately half maximal by ∼400 ms post-cue. Likewise, both samples demonstrate a perisaccadic burst of activity beginning ∼200 ms prior to and peaking around the time of saccade onset. Qualitatively, it is apparent that, in young-stage animals, the cue-related response is slightly stronger whereas the saccade-related activation is both weaker and later-developing (Figure S1). Notably, quantification of these evoked activities (Methods) indicates that, in the young monkeys, the response to the cue in the RF is, on average, 7.9 spikes/s larger than the response associated with a movement into the RF, whereas in the adult monkeys the average difference is only 3.3 spikes/s (*p* = 0.002, permutation test). This suggests that, in contrast to the former sample where the cue representation is more prominent, in the latter the cue and target representations are more balanced. Furthermore, the respective timings of these cue- and saccade-related activations suggests that, during performance of the antisaccade task, opposing cue and goal representations must be simultaneously present (in opposite hemispheres) over the entire range of rPTs leading to asymptotic performance. Thus, to the extent that the relative strength of these conflicting spatial pointers influences antisaccade performance, we would predict (1) a trend from a cue-favoring to a goal-favoring representation with increasing rPT, and (2) a more robust trend for adult-stage than for young-stage animals.

**Figure 6:**
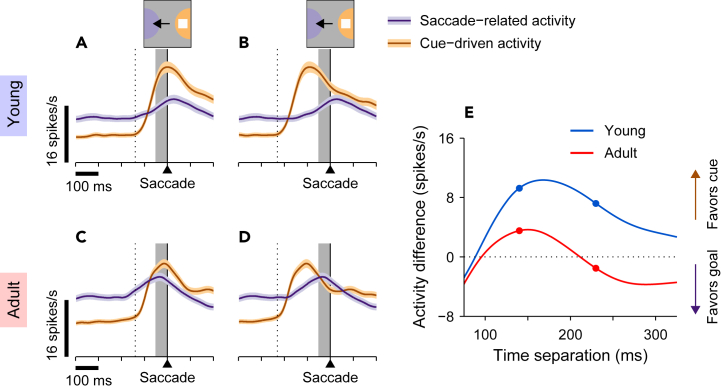
Predicted spatial conflict during antisaccade trials (A–D) Traces show expected cue-driven (brown) and saccade-related responses (purple) obtained by assuming that their magnitudes and latencies will be the same as in the ODR task but that they will overlap in time depending on the interval between cue onset and saccade onset (i.e., the rPT). For the young monkeys (A and B), the expected presaccadic responses are the same as the presaccadic response in Figure 5A (right side, magenta trace aligned to saccade onset), whereas the cue-driven responses are the same as the activity evoked by the cue in Figure 5A (left side, magenta trace aligned to cue onset) but shifted in time. The graphs show the two responses superimposed when the cue onset occurs 140 ms (A, dotted line) or 230 ms (B, dotted line) before the saccade. For the adult monkeys (C and D), the expected responses were constructed in the same way but based on the corresponding ODR activity recorded in the adult sample (Figure 5B, magenta trace). (E) Expected spatial bias as a function of the time separation between cue onset and saccade onset (i.e., rPT). Values on the y axis correspond to the average difference between superimposed cue-driven and saccade-related responses calculated in a 50 ms presaccadic window (gray shades in A–D). Positive (negative) values indicate a cue-driven response that is stronger (weaker) than the saccade-related activity. The four highlighted data points correspond to the examples in panels A–D.

To render intuition about how such neural representations would evolve, we simulated an rPT-dependent interhemispheric competition between cue-favoring and goal-favoring responses based on the activity recorded during performance of the ODR task (Figure 6; Methods). For this, it is important to recall that the ODR requires memory-guided saccades, so the evoked saccade-related activity is endogenously driven. The hypothesis underlying these simulations is that, during antisaccade trials, the cue- and saccade-related activations are essentially the same as those evoked during the ODR task, but (1) are generated by competing populations in the two hemispheres, and (2) are triggered at various times relative to each other because the interval between cue and saccade is no longer fixed. Thus, the two evoked response profiles used in the simulations (Figure 6) were always the same, but the overlap between them varied depending on the temporal separation between the cue and saccade onset, i.e., the rPT. In practice, the cue-driven response (Figures 6A–6D, brown traces) simply shifted in time (compare A vs. B, and C vs. D). For each simulated temporal separation (rPT), the predicted spatial signal was considered equal to the difference between the cue- and saccade-related firing rates evoked during the 50 ms window immediately preceding saccade onset (Figures 6A–6D, shaded areas). Then, by systematically varying the rPT and computing the resulting spatial signal at each point, a full neurometric curve was generated (Figure 6E). Separate predictions were produced for the young (Figures 6A and 6B, blue curve in E) and adult (Figures 6C and 6D, red curve in E) samples based on their corresponding ODR responses. All this involved correct trials only.

The simulations predicted both a transition toward a goal-favoring representation as a function of rPT and differences in the strength of this transition between young- and adult-stage animals. Consider the cue and saccade-related activations (based on the ODR) aligned to simulate the short (Figures 6A and 6C) and long (Figures 6B and 6D) rPTs that correspond to the pre- and post-asymptotic phases of the antisaccade tachometric curve. For short rPTs (Figures 6A and 6C), activity in the 50 ms preceding saccade onset (shaded) strongly favors the cue (brown trace) over the goal (purple trace), with this cue-related bias more pronounced for young- (Figure 6A) than for adult-stage (Figure 6C) animals. For long rPTs (Figures 6B and 6D), the presaccadic representation of the cue (brown trace) weakens while that for the goal strengthens (purple trace), but notably, only in adult-stage animals does the balance shift fully to favor the goal over the cue (Figure 6E, note negative values of red curve at long time separations).

**Figure 7:**
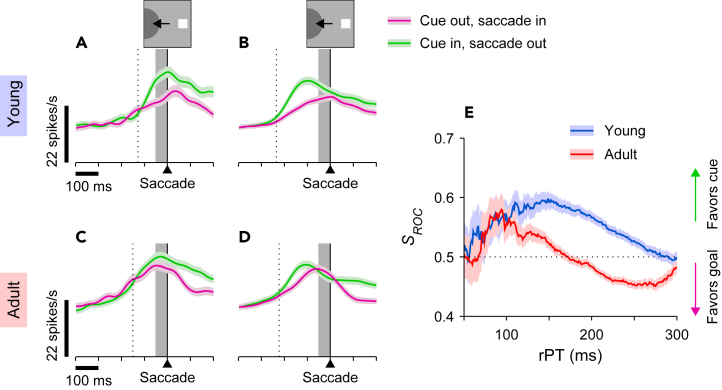
The spatial signal from prefrontal neurons during antisaccade performance varies as a function of processing time and changes between young and adult stages Data are from the same neurons in Figure 5 (256 from young, 338 from adult monkeys) but during performance of the antisaccade task. (A–D) Traces show mean firing rates (±1 SE across neurons) as functions of time for correct saccades into the RF (cue out, saccade in; magenta) or away from the RF (cue in, saccade out; green). Results are for the young- (A and B) and adult-stage (C and D) samples, with trials sorted by rPT into a short range (A and C, 70 ≤ rPT <170 ms) and a long range (B and D, 170 ≤ rPT ≤300 ms). A dotted line marks the median cue onset time in each case. (E) Neurometric curves showing the spatial signal from the recorded neurons (*S*_*ROC*_) as a function of processing time (rPT). The *S*_*ROC*_ is based on the spike counts measured in a 50 ms presaccadic window (gray shades in A–D) across trials, and is defined so that values >0.5 (<0.5) correspond to stronger (weaker) activity toward the cue than toward the saccade location (Methods). Curves are shown for the young (blue; same neurons as in A and B) and adult datasets (red; same neurons as in C and D). Each point represents the *S*_*ROC*_ computed from all the trials within an rPT bin of 80 ms, and shaded error bands indicate 68% CIs (from bootstrap). Data in all panels are from correct trials.

During antisaccade performance, the observed differences between short and long rPTs and between young- and adult-stage samples were qualitatively similar to those simulated on the basis of ODR activation from the same neurons (Figure 7). As predicted, adult-stage neural activity shows clear evidence of a transition from a cue-dominant to a goal-dominant representation. At short rPTs (Figure 7C), activity just prior to saccade onset definitely favors the cue (green trace), whereas at long rPTs (Figure 7D), a reversal of this relationship results in presaccadic activity that now slightly favors the goal (magenta trace). By comparison, any such trend for the young stage (Figures 7A and 7B) is muted, with presaccadic activity continuing to favor the cue (green trace) over the goal (magenta trace) even at long rPTs (Figure 7B).

The neurometric curve based on receiver operating characteristic (ROC) analysis (Methods) reveals this trend as a continuous function of rPT, and comparison between neurometric curves further quantifies differences across developmental stages (Figure 7E). The neurometric curve represents differences in the distributions of spike counts associated with the cue (obtained in cue-in-RF trials) versus the goal (obtained in saccade-into-RF trials) based on spikes counted within the 50 ms interval preceding saccade onset (Figures 7A–7D, shaded areas). The ROC measure on the y axis (*S*_*ROC*_) is defined so that values > 0.5 (< 0.5) correspond to stronger (weaker) activity toward the cue than toward the goal.

For the adult stage, the neurometric curve (Figure 7E, red trace) shows an rPT-dependent progression that strongly resembles the one observed psychophysically (Figure 3B). Specifically, the cue representation rises rapidly to reach a peak near rPT ≈ 100 ms and then declines to transition to a goal-dominant representation beyond rPT ≈ 160 ms. This pattern of rPT dependence is quite consistent with that observed for antisaccade behavior: the rPT ranges corresponding to stronger cue- and goal-related activation generally agree with those likely to produce exogenous capture and asymptotic performance, respectively. For the young-stage, the neurometric curve (Figure 7E, blue trace) also begins with the rapid advance of cue dominance for the range of rPTs where exogenous capture is highly likely but, consistent with the greater susceptibility to capture and poorer asymptotic performance observed at this stage (Figure 3B), a strong cue-related representation persists longer, declines more gradually, and fails to complete the transition to a goal-favoring representation at long rPTs. These results were largely consistent with the simulated responses based on the ODR data (Figure 6E).

**Figure 8:**
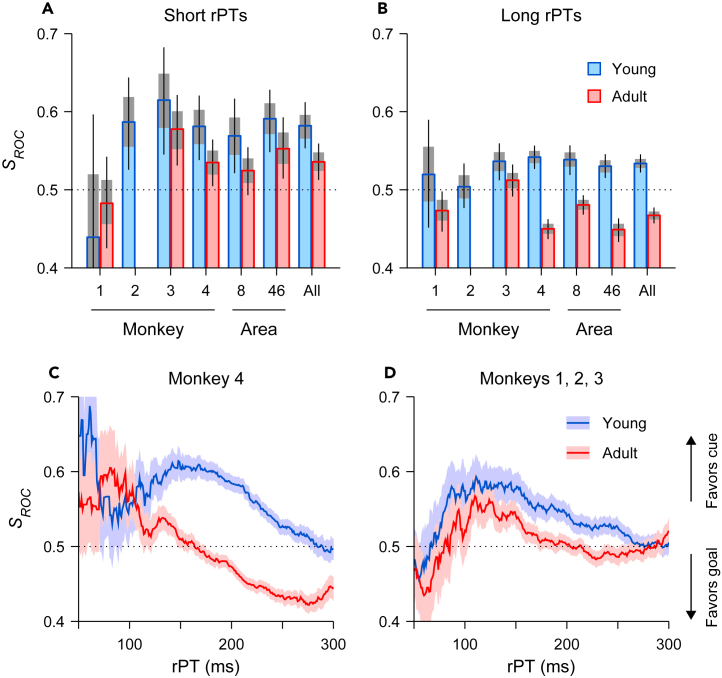
Changes in the spatial signal from prefrontal neurons after maturation are consistent across monkeys and cortical areas Data are from the young (256 neurons, blue bars) and adult samples (338 neurons, red bars) of VM neurons recorded during performance of the antisaccade task. As in Figure 7E, the spatial signal (*S*_*ROC*_) from the recorded neurons was based on the spike count measured in a 50 ms presaccadic window in each trial, but here trials were pooled into two rPT groups only, short and long. (A) Results for short processing times (70 ≤ rPT <170 ms) are shown for each monkey (1–4), for each cortical area (8a or 46), and for all the neurons (All). Bars show *S*_*ROC*_ values for the young (blue) and adult (red) stages, and gray shades and error bars indicate 68% and 95% CIs, respectively, obtained by bootstrapping (Methods). (B) As in A, but for long processing times (170 ≤ rPT ≤300 ms). Note that no VM neurons were recorded from monkey 2 as an adult, and that few trials (51) were available from young monkey 1 at short rPTs. (C) Neurometric curves based on VM neurons recorded from monkey 4. (D) Neurometric curves based on VM neurons recorded from monkeys 1, 2, and 3 combined. *S*_*ROC*_ values in C, D were computed as described for Figure 7E.

Qualitatively, differences in the robustness of the transition from cue to goal representation for the young and adult stages were generally consistent across animals (Figure 8) and for analyses based on recordings from different subregions of prefrontal cortex (Figures 8, S2A, and S2B). For all possible comparisons between conditions with sufficient data (Figures 8A and 8B), *S*_*ROC*_ values indicated a stronger rPT-dependent transition from cue-toward goal-favoring neural signaling in adult-stage animals. For instance, for the data pooled from all the monkeys (Figures 7E, 8A, and 8B, bars labeled “All”), in the young stage, the *S*_*ROC*_ went from 0.58 with a 95% CI of [0.55, 0.61] for short rPTs to 0.53 in [0.52, 0.55] for long rPTs (*p* = 10^−3^ for the difference, from resampling test), whereas in the adult stage the *S*_*ROC*_ went from 0.54 in [0.51, 0.56] for short rPTs to 0.47 in [0.46, 0.48] for long rPTs (*p* < 10^−6^). Differences between young- and adult-stage neurometric functions were particularly evident for monkey 4 (Figures 8C, S2C, and S2D), which contributed the greatest number of neurons to the overall sample (Table 1). When this animal was young, its *S*_*ROC*_ went from 0.58 in [0.53, 0.62] for short rPTs to 0.54 in [0.53, 0.56] for long rPTs (*p* = 0.05), and when it was an adult, its *S*_*ROC*_ went from 0.53 in [0.51, 0.56] for short rPTs to 0.45 in [0.44, 0.46] for long rPTs (*p* < 10^−6^). A similar, albeit attenuated, age-related trend is apparent for the neurometric functions based on data from the remaining three subjects combined (Figure 8D) or from monkey 3 alone (Figures S2E and S2F). When this animal was young, its *S*_*ROC*_ went from 0.61 in [0.55, 0.68] for short rPTs to 0.54 in [0.51, 0.56] for long rPTs (*p* = 0.02), and when it was an adult, its *S*_*ROC*_ went from 0.58 in [0.53, 0.62] for short rPTs to 0.51 in [0.49, 0.53] for long rPTs (*p* < 0.005). For all data subsets, observed differences between the young and adult-stage neural trajectories suggest that endogenously mediated goal-related activation develops earlier and more robustly in the adult-stage sample (see discussion).

**Table 1:** Sample of recorded neurons by area and monkey

	monkey 1	monkey 2	monkey 3	monkey 4	total
**Young sample**					

area 8a	9	7	0	85	101
area 46	0	38	67	50	155
total	9	45	67	135	256

**Adult sample**					

area 8a	47	0	59	98	204
area 46	12	0	22	100	134
total	59	0	81	198	338

Numbers of visuomotor (VM) neurons recorded in each monkey (1–4) and each area (8a or 46) at each stage of development (young or adult). All neurons were recorded during both the ODR and antisaccade tasks.

### Further evidence of PFC participation

The behavioral and neuronal metrics showed parallel rPT-dependent trajectories reflecting the relative strengths of exogenous (cue-related) and endogenous (goal-related) contributions to performance. However, as noted previously, the derived neurometric functions (Figure 7E) reflect a contrast of correct trials only, trials in which either the cue (cue in/saccade out) or the goal (cue out/saccade in) was aligned with the RF of each neuron. As such, it is clear that the saccadic choice cannot be based solely on a direct readout of this prefrontal-based spatial signal. First, at short rPTs, the spatial signal (*S*_*ROC*_) points toward the cue even though saccades were correctly directed toward the antisaccade goal; and second, at long rPTs, the spatial signal does point to the goal but only in the adult stage, and in that case the computed *S*_*ROC*_ values are not commensurate with the observed high levels of behavioral performance. The relatively weak relationship between prefrontal activity and saccadic output is not necessarily unexpected, however, given that putative correlates of attention can be dissociated from saccadic choices.^33^^,^^37^^,^^53^^,^^54^^,^^55^ Seeking further clarification, we searched for additional evidence of prefrontal involvement in antisaccade performance.

**Figure 9:**
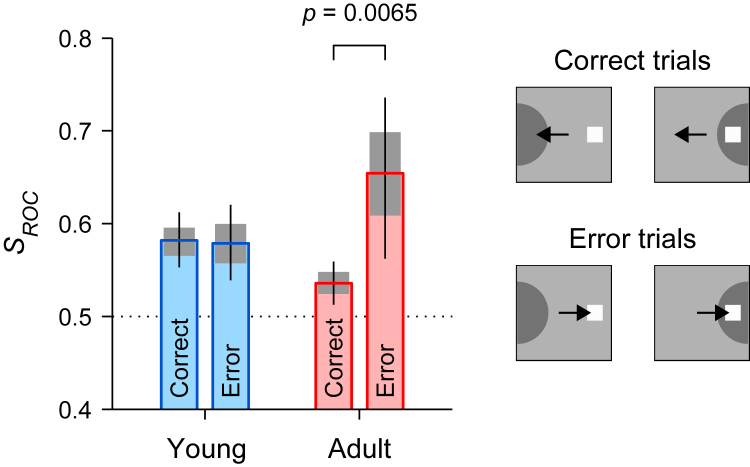
Incorrect saccades likely captured by the cue are associated with stronger cue-related activity in PFC VM neurons The spatial signal (*S*_*ROC*_) observed during short processing times (70 ≤ rPT <170 ms) was separately computed for correct saccades away from the cue (Correct) and for erroneous saccades toward the cue (Error). Results are shown for the young (blue) and adult (red) samples. Gray shades and error bars indicate 68% and 95% CIs, respectively, obtained by bootstrapping. In the adult monkeys, the activity evoked during errors in the capture range is more strongly biased toward the cue than during correct trials (significance based on the bootstrapped distributions). Icons show RFs (dark partial circles) of neurons that compete during correct (top) and error trials (bottom).

For adult-stage monkeys only, we did observe a veridical relationship between neural activity and the probability of making a successful choice (Figure 9). For this contrast, *S*_*ROC*_ values were computed separately for correct and error trials within the “capture” range of rPTs (70 ≤ rPT <170 ms), in which monkeys were most likely to make an error by looking at the cue. Thus, this analysis parses the cue dominance observed for this range (Figures 7A–7C and 7E) to test the hypothesis that a stronger (weaker) cue-pointing spatial signal is associated with a greater (lower) likelihood of exogenous capture. For adult-stage animals (Figure 9; red bars), this was clearly the case: erroneous captured saccades occurred in association with greater neural bias toward the cue than did correct saccades in the same rPT range. For young-stage animals (Figure 9, blue bars), no such relationship was apparent, with the cue-related bias indistinguishable for correct and error trials. These results suggest that the dlPFC and FEF make a stronger contribution to antisaccade performance in adults than in young monkeys.

## Discussion

We examined antisaccade behavior and associated prefrontal activity of developing monkeys before and after their transition to adulthood. With insights derived from recent human studies,^50^^,^^51^ the present findings assess antisaccade performance with respect to spatial attentional control. In doing so, the findings reveal how extrinsic and intrinsic factors interact dynamically to determine monkey antisaccade performance, how changes in this interaction relate to developmental improvements in this ability, and how parallel changes in prefrontal neural representations could contribute to maturing behavioral capabilities.

### Maturation of antisaccade performance reflects the changing dynamics of exogenous and endogenous control

Though termed “response inhibition”, the ability to suppress an inappropriate response to a prepotent stimulus to prioritize task relevant behavior relies on the interaction of multiple mental operations encompassing sensory, motor, and cognitive processes.^18^^,^^19^^,^^40^^,^^41^^,^^51^^,^^56^^,^^57^ To gain deeper insight into how these capacities mature in the service of improved behavioral control, we evaluated both antisaccade behavior and associated neural activity within a framework in which the contributions of exogenous and endogenous factors to performance can be resolved with high temporal precision as they evolve during a visuomotor choice.^50^^,^^51^^,^^58^

The antisaccade task requires visual stimulus detection (an exogenous process) followed by application of the antisaccade rule (an endogenous process) to plan a motor response to look away.^2^ In agreement with recent results from human participants,^50^^,^^51^ we found that monkey antisaccade behavior can be characterized in large part as a time dependent competition between early acting exogenous and later developing endogenous mechanisms of spatial attentional control. Our analyses revealed a pattern of monkey antisaccade performance that closely mirrored that of human subjects. Specifically, the tachometric curves for monkey subjects demonstrated a rapid and precipitous decline in accuracy for saccades generated at short (∼100 ms) cue viewing times, when subjects were unlikely to resist the exogenous pull of the salient cue. With additional rPT, the likelihood of exogenous capture quickly receded and performance recovered to progress toward an asymptote. Both young and adult monkeys followed the same overall pattern. However, substantial differences were evident between developmental stages in the relative timing and potencies of the exogenous and endogenous contributions: capture by the salient cue was more transient, and the rise toward asymptotic performance more rapid, for adult-stage animals. Adult monkeys also achieved higher levels of asymptotic performance. This effect was more subtle, yet consistent, and suggests that adult-stage animals were also less prone to lapses in performance and more reliable in their ability to apply the task rule. Thus, we were able to further distinguish between factors that depend on processing time (exogenous capture and endogenous recovery) and those that do not.

### Neural signatures of the exogenous-endogenous competition parallel developmental changes in antisaccade behavior

Prominent early models suggested that dlPFC suppresses reflexive responding on the antisaccade task by providing an inhibitory signal to ipsilateral structures more proximal to motor output.^2^^,^^21^^,^^22^^,^^23^^,^^24^ However, this view is challenged by more recent findings demonstrating that dlPFC exerts a net excitatory effect on projection targets such as the ipsilateral superior colliculus (SC).^25^^,^^26^^,^^27^ These empirical data are consistent with modeling accounts of antisaccade performance that do not invoke an inhibitory “stop” process, but instead consider a competition between motor alternatives driven by sensory (exogenous) and rule-based (endogenous) signals.^50^^,^^51^^,^^58^^,^^59^^,^^60^ Thus, rather than actively suppressing a response to the cue, prefrontal activity positively biases the competing downstream representations, such as those corresponding to SC motor plans for cue and antisaccade goal, with antisaccade performance determined by which of these motor plans first reaches the threshold for triggering a saccade. Such is generally consistent with the view that VM activity in dlPFC and FEF contributes to a larger network for biasing spatial attention according to both bottom-up (exogenous) and top-down (endogenous) factors.^48^^,^^61^^,^^62^^,^^63^^,^^64^^,^^65^^,^^66^^,^^67^^,^^68^^,^^69^^,^^70^^,^^71^ Interpreted in this context, in which attention mechanisms generally contribute to resolving a target selection conflict,^37^^,^^46^^,^^49^^,^^58^^,^^72^^,^^73^ the present findings provide a parsimonious account for how changes in PFC activity could relate to developmental improvements in antisaccade performance.

In adult-stage monkeys, two observations suggest that the mature PFC plays a role in response control. First, the PFC spatial signal and behavioral accuracy followed consistent trajectories as functions of rPT (Figures 3B and 7E, red traces). Specifically, the visual cue representation dominated over the range of rPTs likely to result in exogenous capture, whereas the representation of the antisaccade goal dominated over the range of rPTs in which performance was endogenously driven. These parallel transitions are strongly suggestive because they are so rapid, the difference between maximum capture and asymptotic performance being 50–100 ms of processing time. The second observation indicative of a direct contribution of PFC to adult antisaccade performance was that a spatial signal more strongly favoring the cue was associated with an increased probability of making an incorrect saccade to the cue (Figure 9, red bars).

In young-stage monkeys, the correlation between neural activity and behavior was less definitive: the representation of the antisaccade goal again increased in strength with a similar time course as choice accuracy, but it did not ultimately overcome the cue representation even at long rPTs (Figure 7E, blue trace), when the animals were consistently performing near 90% correct (Figures 3B and 4A, blue traces). Furthermore, no association between the spatial signal and trial outcome was detected for the range of short rPTs most likely to produce saccadic capture (Figure 9, blue bars).

Thus, tighter and looser correlations between neural activity and behavior were observed for adult and young monkeys, respectively. Such changes across developmental stages are also consistent with the differences observed in behavior, with the influence of the cue more short-lived and the representation of the goal more robust in mature animals. Given these findings, the PFC activity recorded during antisaccade performance can be interpreted at face value, i.e., simply as a superposition of exogenous and endogenous signals from opposite hemispheres that is generally tilted toward the former, but more so at the young stage. The close correspondence between the observed results (Figure 7) and those predicted on the basis of ODR activity (Figure 6) supports this view. Such correspondence implies that the VM neurons in PFC are fundamentally communicating the occurrence of the cue and of the endogenously driven saccade via signals that evolve with stereotypical time courses regardless of the task. More complex interactions or dependencies are unnecessary for explaining the activity evoked during antisaccade trials. In this regard it is worth noting that, in both tasks, differences in the strength of cue encoding between young and adult-stage animals were modest, whereas earlier and more robust signaling of the goal was readily apparent (Figure S1). Again, taken at face value, this would suggest a greater impact of development on the ability to reorient spatial attention endogenously rather than on the ability to suppress the exogenously driven response.

### Prefrontal attention dynamics must act in coordination with other areas

Our findings outline a functional role for dlPFC and FEF in antisaccade performance as it matures, namely, providing a time-dependent readout of spatial-attention pointers. However, they do not (and would not be expected to) provide a complete neural account of a subject’s behavior. The formation of a robust goal representation most likely engages multiple brain regions; when saccades are involved, at least the lateral intraparietal area^61^^,^^74^^,^^75^^,^^76^^,^^77^ and the SC^78^^,^^79^^,^^80^^,^^81^ would be expected to also contribute to resolving any competition between exogenous and endogenous signals. For example, in adult animals, neurophysiological studies of the lateral intraparietal area have emphasized its role in signaling stimulus salience,^75^ and have noted differences in the relative strengths of stimulus versus goal representations during antisaccade performance.^76^^,^^82^ Notably, in terms of development, the observed changes in PFC neural dynamics are most likely part of a system-wide refinement of neural circuits that support increased cognitive and visuomotor capabilities.^6^^,^^14^^,^^15^^,^^83^

Beyond the fact that many visuomotor areas have been implicated in attention-related signaling, two observations in particular underscore the necessary participation of other areas. First, one might wonder why, in young-stage animals, the spatial signal never pointed to the goal, even when the monkeys performed most trials correctly (Figure 7E, blue trace). During antisaccade trials, some neurons will convey exogenous information exclusively (e.g., purely visual neurons), some will encode the endogenous goal only (e.g., purely motor-related neurons), and others, like the recorded VM neurons, will convey both in varying proportions.^11^^,^^33^^,^^84^ All such neurons may play a causal role. The recorded VM neurons happen to convey the information related to cue location somewhat more robustly than that related to goal location, so their spatial signal is generally biased toward the former, particularly in the young-stage animals. What is notable is that their spatial signal *shifts* in a way that parallels behavioral shifts across both short (single-trial) and long (developmental) timescales. But, clearly, the VM activity offers only a limited and biased view of the circuit dynamics.

Second, why did the spatial signal in the young-stage animals show no apparent impact on trial outcome (Figure 9, blue bars)? One possibility is that the influence of PFC on performance becomes effective only once the maturation process has advanced beyond a certain point. This would be surprising, given that the cue- and saccade-related responses are already found in the structure, but since our study is purely correlational, we cannot unequivocally rule out this scenario. Another possibility is that the influence of PFC on performance exists in young-stage animals but is weaker than in adults, simply too weak to be detected given the numbers of trials and neurons we collected. Either way, the result means that other neurons must be more strongly dictating the outcome of the task in that case.

### Conclusion

The present findings demonstrate an entirely novel aspect of the developmental trajectory that leads to improved response control in primates. By conceptualizing antisaccade performance as a time-varying competition between exogenous and endogenous mechanisms, and resolving their contributions with millisecond precision, we were able to show that alterations in this temporal dynamic account for key aspects of the transition to adult-like performance. Moreover, the behavioral findings are parsimonious with well-established roles of prefrontal visuomotor activity in the deployment of both bottom-up (exogenous) and top-down (endogenous) spatial attention. Finally, we note that the framework based on attention and processing time may generalize as an aid to revealing the neural substrates for differences in antisaccade performance more generally, such as those associated with impaired cognition consequent to disease^85^^,^^86^^,^^87^ or normal aging.^9^^,^^88^^,^^89^

### Limitations of the study

The present findings clearly point to changes in PFC activity as a correlate of cognitive maturation, yet it is also clear, as discussed previously, that the PFC neurons under study here can only be part of the substrate for improved behavioral control. Thus, our study cannot speak to the strength of any such causal contribution. Similarly, we infer from the tachometric curve that an rPT-dependent shift in spatial attention occurs as an antisaccade trial unfolds. Although this is a logical inference, and one amply supported by decades of research on exogenous and endogenous attention, the study design did not permit independent assessment of the strength of attentional deployment at cue or goal locations (e.g., via measurement of perceptual acuity^53^). A second consideration is that the longitudinal design, while essential to our ability to track age-related changes neurophysiologically, allows for the possibility that factors other than cognitive maturation, such as prior task exposure and/or memory consolidation, contributed to observed behavioral and neurophysiological differences between young- and adult-stage animals. Although impossible to rule out definitively, we sought to minimize the influence of session-to-session practice (see STAR Methods) and note that the benefits of offline procedural memory consolidation for simple visuomotor tasks is typically reported to occur on the timescale of hours or days,^90^^,^^91^^,^^92^^,^^93^^,^^94^^,^^95^^,^^96^^,^^97^ not a year or more as was the behavioral hiatus period in the current study.

## STAR★Methods

### Key resources table

**Table:** 

REAGENT or RESOURCE	SOURCE	IDENTIFIER
**Software and algorithms**		

MATLAB and Statistics Toolbox	Mathworks	https://www.mathworks.com/products/matlab.html
WaVE data acquisition	Meyer and Constantinidis^98^	https://www.sciencedirect.com/science/article/pii/S0165027004002535?via%3Dihub
Behavioral data analysis	Salinas et al.^51^	https://elifesciences.org/articles/46359

### Resource availability

#### Lead contact

Further information and requests for resources should be directed to and will be fulfilled by the lead contact, Terrence R. Stanford: stanford@wakehealth.edu.

#### Materials availability

This study did not generate new unique reagents.

#### Data and code availability

• All data reported in this paper will be shared by the lead contact upon request.
• This paper does not report original code
• Any additional information required to reanalyze the data reported in this paper is available from the lead contact upon request.

### Experimental model and study participant details

Four male rhesus monkeys (*Macaca mulatta*) were used in this study as described in a previous report.^10^ Briefly, we obtained morphometric measures (body weight, crown-to-rump length, chest circumference, ulna and femur length, testicular volume, eruption of canines), determined bone maturation by X-rays of the upper and lower extremities, and assayed serum concentration of circulating hormones, including testosterone [T] and dihydrotestosterone [DHT]. Using these measures, we determined when monkeys entered puberty. Based on this reference, we collected behavioral data in mid-adolescence and in adulthood, 1.6–2.1 years later. Median age was 4.3 years at the adolescent stage (range: 4.0–5.2 years) and 6.3 years at the adult stage (range: 5.6–7.3). All surgical and animal use procedures were reviewed and approved by the Institutional Animal Care and Use Committee of Wake Forest School of Medicine (protocols A12–009, A14-214), in accordance with the U.S. Public Health Service Policy on humane care and use of laboratory animals and the National Research Council guide for the care and use of laboratory animals.

### Method details

#### Behavioral tasks

Monkeys were trained to perform an oculomotor delayed response (ODR) task and three variants of the antisaccade task (Figure 2) during the young stage. Data collection for the evaluation of young-stage behavioral performance occurred over a period of 1–3 quarters and, for each animal. To minimize the impact of session-to-session practice effects, recording commenced only after initial training had yielded stable task performance. On average, young-stage naive animals required 43 sessions to achieve consistent session-to-session performance. At the conclusion of young-stage data collection, animals were returned to their colony and were no longer tested or trained for a period of ∼1 year. Thereafter, adult-stage behavioral and neurophysiological evaluations commenced. On average, 17 behavioral sessions were needed to reacclimate adult animals to the task, at which time session-to-session performance level was stable.

In the antisaccade task, each trial starts with the monkey fixating on a central green point on the screen. After fixating for 1000 ms, the cue is presented for 100 ms, consisting of a 1° white square stimulus that may appear at one of eight locations arranged on a circle of 10° eccentricity. The monkey is required to make a saccade to the location diametrically opposed to the cue. For a reward to be delivered, the saccade must terminate within a 5–6° radius window centered on this location and the monkey must hold fixation within this window for 100 ms.

We used three variants of the antisaccade task: overlap, zero gap, and gap. These differed in the timing of the cue onset relative to the fixation point offset (Figure 2A). In the overlap condition, the cue appears first and then the fixation point and cue are simultaneously extinguished. In the zero-gap condition, the fixation offset and the cue onset occur at the same time. In the gap condition, the fixation turns off and the screen remains blank for 100 ms before the cue onset (in some experimental sessions, an additional gap condition with a 200 ms blank was used). The monkeys were trained in the antisaccade task with the stimulus appearing at one of eight possible stimulus locations chosen pseudo-randomly. However, in approximately 75% of the trials the stimulus appeared at a cardinal location (left, right, up, or down), whereas in the remaining 25% of the trials it appeared at one of the four diagonal locations. These proportions were similar in the young and adult stages. In order to prevent the monkeys from developing biases or aborting some trial types altogether, the correct completion of a block of trials involving 4 randomized cue locations x 3 task variants was required before initiating the next randomized block of trials. No substantial differences in performance were found between cardinal and diagonal conditions, so the datasets were combined.

In addition to the antisaccade task, the monkeys performed the ODR task, which requires subjects to remember the location of a cue stimulus. Each ODR trial starts with the monkey fixating a central green point. After 1000 ms of fixation, a 1° white square stimulus is presented for 500 ms at one of eight locations arranged on a circle of 10° eccentricity. After a 1500 ms delay period, the fixation point is extinguished, instructing the monkey to make an eye movement to the remembered location of the cue within 600 ms. The ODR task was used for determining the cells’ response fields (RFs) and for dissociating their visually driven (after cue onset) and motor-related activities (before saccade onset).

Animals were rewarded with fruit juice for successful completion of a trial. Eye position was sampled at 240 Hz with an infrared eye tracking system (ISCAN, RK-716; ISCAN, Burlington, MA). Breaking fixation at any point before the offset of the fixation point aborted the trial and resulted in no reward. For non-aborted trials, the time of saccade onset was determined based on a joint spatial and velocity criterion. For displacements of eye position that carried the eyes beyond the fixation window (1.5°–2.8° radius), saccade onset was defined as the time point at which saccade speed exceeded 100°/sec. The display of visual stimuli and monitoring of eye position were controlled with in-house software^98^ implemented in the MATLAB environment (Mathworks, Natick, MA) and utilizing the Psychophysics Toolbox.^99^

#### Neurophysiological recordings

After each young animal reached asymptotic performance in the behavioral tasks, a 20 mm diameter recording cylinder was implanted over its prefrontal cortex. Localization of the recording cylinder and of electrode penetrations within the cylinder was based on MR imaging processed with the BrainSight system (Rogue Research, Montreal, Canada). Recordings were collected with epoxylite-coated Tungsten electrodes with a diameter of 250 μm and an impedance of 4 MΩ at 1 kHz (FHC, Bowdoin, ME). Electrical signals were amplified, band-pass filtered between 500 Hz and 8 kHz, and stored through a modular data acquisition system at 25 μs resolution (APM system, FHC, Bowdoin, ME). Recorded spike waveforms were sorted into separate units using an automated cluster analysis method based on the KlustaKwik algorithm.^100^ Recordings analyzed here were obtained from areas 8a and 46 of the PFC. As described above for the acquisition of behavioral data, a first round of single-unit recordings took place during the young stage; then, following a period of ∼1 year during which the monkeys did not perform any tasks, another round of recordings from the same areas was completed, using identical recording methods, after the animals had reached adulthood (for further details, see^10^^,^^11^).

#### Neuron selection

We classified PFC neurons as visual, motor, or VM based on whether activity was temporally linked to the occurrence of the sensory stimulus, the saccade, or both of these events during performance of the ODR task.^11^ Our classification scheme is directly analogous to those made on the basis of visually guided saccade tasks.^33^^,^^84^ Here, no assumptions are made about how the prefrontal population is decoded downstream, but only VM neurons are included in the analysis (Table 1) to ensure that the comparison of cue and goal representations across age groups is not biased by an over- or under-abundance of either purely visual (cue) or purely motor-related (goal) neurons. This way, a faster transition from cue-to goal-related representations in adults, for example, cannot be due to biased sampling of visual versus motor cells. VM neurons are the most common, and because each one contributes to both representations, such a transition must reflect a change in the vigor or timing of the activity linked to cue and saccade onsets.

For each neuron, the RF was first determined based on the responses in the ODR task.^10^^,^^11^ The mean firing rate during the 500 ms of cue presentation was compared to that in the 1000 ms fixation period preceding it (paired t-test, *p* < 0.05), and the RF was the best cue location given this criterion. Neurons that did not demonstrate a significantly elevated firing rate at any cue location were excluded from analysis. In addition to visual responsiveness, a second criterion for inclusion was significant presaccadic activation. That is, the mean firing rate in the 250 ms response period after the go signal (fixation offset) had to exhibit a significant increase over the baseline fixation period (paired t-test, *p* < 0.05), for at least one stimulus location. Finally, to be included in the analysis, a neuron had to respond in qualitatively similar fashion on the antisaccade task, as evidenced by significantly elevated mean firing rate in the 250 ms window following the onset of the cue in the RF as compared to the 1000 ms fixation interval. This reevaluation of the visual activity served as a consistency check to validate that the same neuron remained isolated during data collection for the antisaccade task.

All the neurons thus selected were considered responsive and to have visually driven and presaccadic activity, and hence could be classified as VM. The VM populations used for analysis (256 and 338 neurons for the young and adult samples, respectively) are broken down by monkey and recorded area in Table 1. The numbers of trials associated with each of these recorded VM neurons were 71 ± 12 (mean ± SD) in the ODR task and 110 ± 21 in the antisaccade task. This corresponds to approximately 9 trials per tested cue location in the ODR task (8 locations recorded per neuron) and 27 trials in the antisaccade (4 locations recorded per neuron), on average. The numbers were nearly identical for the young and adult samples.

To quantify the relative strengths of the cue-driven and presaccadic responses elicited in the ODR task, in each trial in which the cue was presented in the RF, firing rates were computed in two time windows. The first one was from 50 to 150 ms after cue onset, and the second from −100 to 0 ms before saccade onset. The average difference between the two firing rates was then calculated for each analyzed neuron. Finally, the mean differences (contrasting time windows) for the young- (7.9 ± 1.22 spikes/s, mean ± SEM, n = 256 neurons) and adult-stage samples (3.3 ± 0.91 spikes/s, mean ± SEM, n = 338 neurons) were computed along with their overall difference (contrasting developmental stages). The results showed that the response to the cue was stronger than that preceding the saccade in both samples, but the differential was substantially larger for the young monkeys (*p* = 0.002, non-paired, two-sided permutation test).

### Quantification and statistical analysis

#### Behavioral data analysis

We analyzed performance in each variant of the antisaccade task by determining the proportion of trials that resulted in correct responses. We used a spatial criterion (90° window) for these analyses, such that saccades were considered correct if they landed within ±45° of the target direction, incorrect if they were within ±45° of the cue direction, and intermediate otherwise (and were not included in either the correct or incorrect proportion). Importantly, although the absolute percentages of correct and incorrect trials depended on the chosen criterion, the fraction of correct to valid (correct plus incorrect) trials did not. Specifically, the time courses of behavioral performance (detailed below) were qualitatively very similar for a range of criteria going from most permissive (180° window) to extremely strict (11.25° window). The standard criterion (90° window) was used for all analyses except where otherwise indicated.

We used two metrics for determining the speed of the response in the antisaccade task. The reaction time (RT) was defined as the time between fixation point offset, which was the signal for the monkey to initiate a response, and saccade onset. Additionally, the raw processing time (rPT) was defined as the time between the onset of the visual stimulus and the onset of the saccade. Thus, in each trial, the rPT corresponds to the amount of time that the subject had for viewing and processing the visual stimulus before initiating the saccade.^50^^,^^51^^,^^52^^,^^58^ The relationship between performance and rPT is given by the tachometric curve, a psychometric function that is unique in its ability to time resolve the competitive interaction between the exogenously salient stimulus and the endogenously defined goal.

In general, analyses of performance as a function of rPT were the same as in previous studies with human subjects.^50^^,^^51^ We summarize the key procedures here. All data analyses were performed in MATLAB (The MathWorks, Natick MA). First, rPT was computed for each trial. For trials in the overlap condition, rPT=RT+100; for trials in the zero-gap condition, rPT=RT; and for trials in the gap condition, rPT=RT−100 (and rPT=RT−200 for those gap trials in which the blank period lasted 200 ms). To compute the tachometric curve (Figures 3B and 4A), trials were grouped into rPT bins with bins shifting every millisecond. Numbers of correct and incorrect trials were then counted within each bin. From these numbers, we calculated the proportion of correct choices and, using binomial statistics, confidence intervals (CIs) for the proportion. For the curves of individual monkeys (Figure 4A), the rPT bin was 40 ms; otherwise, it was 20 ms.

Three quantities were used to characterize the perceptual performance associated with each tachometric curve (Figure 4B): the rPT at which a criterion of 75% correct was achieved, the probability of capture, and the asymptotic performance level. All three were obtained by first fitting the tachometric curve with a continuous analytical function. The fitting curve was a combination of two sigmoidal functions.^51^ It was defined as

v(x)=max(L(x),R(x),0)

where the maximum function, max(a,b,c), returns the largest of a, b, or c; and L(x) and R(x) are the two sigmoidal functions given by

$$L\left(x\right)=B+\frac{A_{L}-B}{1+\exp\left(\frac{x-C_{L}}{D_{L}}\right)}$$

$$R\left(x\right)=B+\frac{A_{R}-B}{1+\exp\left(-\frac{x-C_{R}}{D_{R}}\right)}$$

where L(x) tracks the left (decreasing) side of the tachometric curve and R(x) tracks the right (increasing) side. The parameter $C_{L}$ is the processing time at which the fraction correct is halfway between chance (equal to $A_{L}$) and minimum (equal to B), whereas $C_{R}$ is the processing time at which the fraction correct is halfway between the minimum and asymptotic (equal to $A_{R}$). The parameters $D_{L}$ and $D_{R}$ determine how rapidly performance falls below chance initially and how rapidly it recovers toward 100% correct later on. For any given tachometric curve, the chance level was fixed at 50% correct by setting $A_{L}=0.5$, and the remaining six parameters were determined by minimizing the mean absolute error between the experimental data and the analytical curve using the MATLAB function fminsearch.

Having fitted this continuous curve to the data, we simply readout from it the rPT at which 75% correct was achieved; the asymptotic performance level was equal to the parameter $A_{R}$; and the probability of capture was computed as follows. Within a fixed rPT range (0–300 ms), we calculated the area between the fitted curve, v(x), and the horizontal line at chance (i.e., at a fraction correct of 0.5). This was done separately for v(x)<0.5, giving rise to an area $A_{under}$, and for v(x)>0.5, giving rise to an area $A_{over}$, both always positive. Then we set

$$P\left(\text{capture}\right)=\frac{A_{under}}{A_{under}+A_{over}}$$

This way, the probability of capture indicates how often performance was below chance; it is equal to 0 if the fitted tachometric curve never drops below chance and to 1 if the curve never rises above chance.

Bootstrapping techniques^101^^,^^102^ were used to generate CIs for these three quantities, as detailed previously.^51^ To do this, the trial-wise data were resampled with replacement, the resulting resampled tachometric curve was re-fitted, and the three curve features of interest (rPT at criterion, probability of capture, and asymptote) were recalculated and stored. This resampling process was repeated 500 times to generate distributions for each of the three quantities of interest. The reported 95% CIs correspond to the 2.5 and 97.5 percentiles obtained from the bootstrapped distributions of values.

#### Neural data analysis

For each neuron, continuous firing rate traces, or spike density functions, were produced by aligning the recorded spike trains to relevant task events (cue onset, saccade onset), convolving them with a Gaussian kernel (σ = 21 ms), and averaging across trials. Population traces were then generated by averaging across cells, with error bands computed as the standard error of the mean (SEM) at each time point. This was done separately for trials that resulted in saccades into the RF (Figures 5 and 7A–7D, magenta traces) and for trials that resulted in saccades diametrically away from the RF (Figures 5 and 7A–7D, green traces). Note, however, that in the ODR task, target and cue locations in correct trials coincide, whereas in the antisaccade task, target and cue locations in correct trials are on opposite hemifields.

To visualize how the neural activity depended on processing time (Figures 7A–7D), trials were parsed into short and long rPT time bins. We considered a range in which saccades were likely to be captured by the cue (short, 70 ≤ rPT <170 ms) and a range in which most saccades were correct (long, 170 ≤ rPT ≤300 ms). These ranges were deliberately broad to demonstrate the effect of processing time on neural activity as plainly as possible. However, the resulting population responses only depended weakly on the exact cutoffs used. To rigorously quantify how the PFC activity depended on rPT, a more detailed analysis using a moving time window was performed. Specifically, we aimed to determine how the spatial location encoded by the PFC population changed as a function of rPT (Figure 7E). This was as follows.

The spatial signal, or *S*_*ROC*_, was computed using standard methods from signal detection theory.^103^^,^^104^ This measure corresponds to the accuracy with which an ideal observer can classify data samples from two distributions and is equivalent to the area under the receiver operating characteristic, or ROC, curve. A value of 0.5 corresponds to distributions that are indistinguishable (chance performance, full overlap), whereas values of 0 or 1 correspond to fully distinguishable distributions (perfect performance, no overlap).

In correct antisaccade trials, the *S*_*ROC*_ was used to quantify the degree to which prefrontal neurons were differentially activated by the cue in the RF (and saccade away) versus by the saccade into the RF (and cue outside). Thus, in this case *S*_*ROC*_ > 0.5 indicates higher activity for cue-in than for saccade-in trials, and *S*_*ROC*_ < 0.5 indicates the opposite, higher activity in the saccade-in than in the cue-in condition. The activity measure used to calculate these *S*_*ROC*_ values was the spike count elicited just prior to choice onset (in a window from −50 to 0 ms aligned on saccade onset; gray shaded areas in Figures 7A–7D), a presaccadic window intended to assess cue versus goal related activity just prior to saccade commitment. In summary, then, a spike count was measured in each trial, trials were sorted into two groups according to cue and saccade locations, and the *S*_*ROC*_ was used to measure the separation between the two resulting spike count distributions.

Continuous neurometric functions comparable to the behavioral tachometric curves (Figure 7E) were generated by first pooling the data across neurons and then calculating *S*_*ROC*_ as a function of rPT; that is, for the trials falling within a given rPT bin (bin width = 80 ms, shifted every 1 ms). The pooling involved two steps. First, the presaccadic spike counts of each neuron were centered by subtracting a constant θ that was cell-specific, and then the centered spike counts from all the neurons were sorted into two groups, for cue-in and saccade-in trials. The *S*_*ROC*_ compared responses from these two pooled distributions within each rPT bin. For each neuron, the constant θ was equal to (μ_cue_ + μ_sac_)/2, where μ_cue_ and μ_sac_ are the mean spike counts for cue-in and saccade-in trials. Centering this way is like subtracting the overall mean spike count of each neuron, but accounting for the fact that the numbers of cue-in and saccade-in trials may be very different. Other normalization schemes produced qualitatively similar trends. It is important to keep in mind that, although we plot the *S*_*ROC*_ as a function of processing time, its value was always based on the spike counts measured just prior to saccade onset.

For any given *S*_*ROC*_ value, an error bar was computed by bootstrapping,^102^^,^^105^ that is, by repeatedly resampling with replacement (1000 iterations) the two underlying data distributions and recomputing the *S*_*ROC*_ each time. From the resulting *S*_*ROC*_ distribution, a 95% CI (containing 95% of the data) and a 68% CI (containing 68% of the data) were calculated. Results in Figures 7 and 8 include these intervals indicated via error bars and error shades, respectively. To avoid crowding due to excessive overlap, Figures 7E, 8C, and 8D only show the 68% CIs for the resampled *S*_*ROC*_ values obtained at each rPT bin. When comparing *S*_*ROC*_ values or other measures of activity across conditions (e.g., Figure 9), significance was established using one- or two-sided permutation tests (for paired data) or equivalent randomization tests (for unpaired data), as applicable.^106^

#### Modeled antisaccade responses

The neural responses recorded in the ODR task were used to generate predictions for the activity to be expected in the antisaccade task just prior to saccade onset (Figure 6). The premise for the predictions is that, during antisaccade trials, the cue-driven and saccade-related responses are the same as those observed in the ODR task, except that (1) they are delivered by different neural populations in opposing hemispheres, and (2) they overlap in time to varying degrees, depending on the temporal separation between cue onset and saccade onset (i.e., the rPT). An underlying assumption or null hypothesis built into this conceptual model is that the two neural signals interact weakly or not at all, so that one cannot substantially suppress the other. In this framework, what matters to downstream networks is the relative intensities of the two signals that point to opposite spatial locations.^61^^,^^74^^,^^107^

Predictions were made for correct antisaccade trials and were generated separately for each developmental stage. For brevity, we describe the implementation for the young sample only, but that for the adult was entirely analogous. First, the expected presaccadic activity in antisaccade trials (Figures 6A and 6B, dark blue traces) was set equal to the response to saccades into the RF during the ODR task (Figure 5A, magenta trace on rightmost axis). Because the time axis was always aligned to saccade onset, this predicted presaccadic response was the same for all trials. Next, the expected cue-related activity in antisaccade trials (Figures 6A and 6B, brown traces) was set equal to the response to the cue appearing in the RF during the ODR task (Figure 5A, magenta trace on leftmost axis). Crucially, however, this predicted activity was presumed to be triggered by the cue onset, so it could start rising anywhere from just before the (anti) saccade, if the interval between cue onset and movement onset was small (short-rPT), to well before the (anti) saccade, if the interval was large (long-rPT). This difference in timing accounts for the different predictions in Figure 6A (rPT = 140 ms) and Figure 6B (rPT = 230 ms). The key quantity to evaluate in each case is the difference in the magnitude of the cue- and saccade-related representations in the 50 ms prior to saccade onset, which is when the responses are most likely to have an impact on the saccadic choice. This difference varies systematically with the interval between the cue and saccade onsets, which is the rPT (Figure 6E).

It should be noted that the predicted responses are anticipated to be wrong in two respects. One is the initial level of activity for the presaccadic response, which is well above baseline during the delay period of the ODR task. This is not a major concern because the full response still clearly develops within the last 100 ms or so before movement onset, peaking within less than 50 ms of it. The other anticipated discrepancy is that the actual activity is likely to change after the saccade; however, this cannot affect the triggered saccadic choice substantially.
