# Supplementary material for: Parallel signatures of cognitive maturation in primate antisaccade performance and prefrontal activity
Source: iScience. 2024 Jul 11;27(8):110488. doi: 10.1016/j.isci.2024.110488 (PMC11326912; doi:10.1016/j.isci.2024.110488)
Supplement: Document S1. Figures S1 and S2 [file mmc1.pdf]

iScience, Volume *27*

Supplemental information

Parallel signatures of cognitive maturation in primate antisaccade performance and prefrontal activity

Junda Zhu, Xin Maizie Zhou, Christos Constantinidis, Emilio Salinas, and Terrence R. Stanford

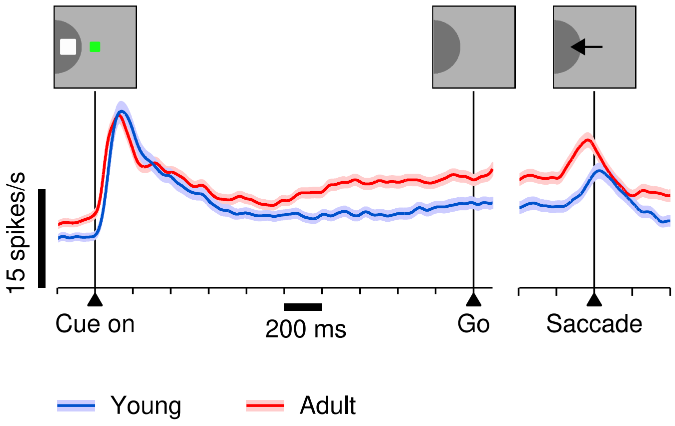

Figure S1. Young- versus adult-stage activity during the ODR task. Related to Figures 5, 6.

Results are from VM neurons recorded in the ODR task in the young- (blue trace) and adult-stage animals (red trace). Activity traces correspond to mean firing rate (± 1 SE across neurons) as a function of time for correct saccades into the RF (cue in, saccade in). The data are the same as the magenta traces in panels A (young animals) and B (adult animals) of Fig. 5, but superimposed. In adults (red trace), higher goal-directed activity is evident during the memory delay and is followed by an earlier perisaccadic burst.

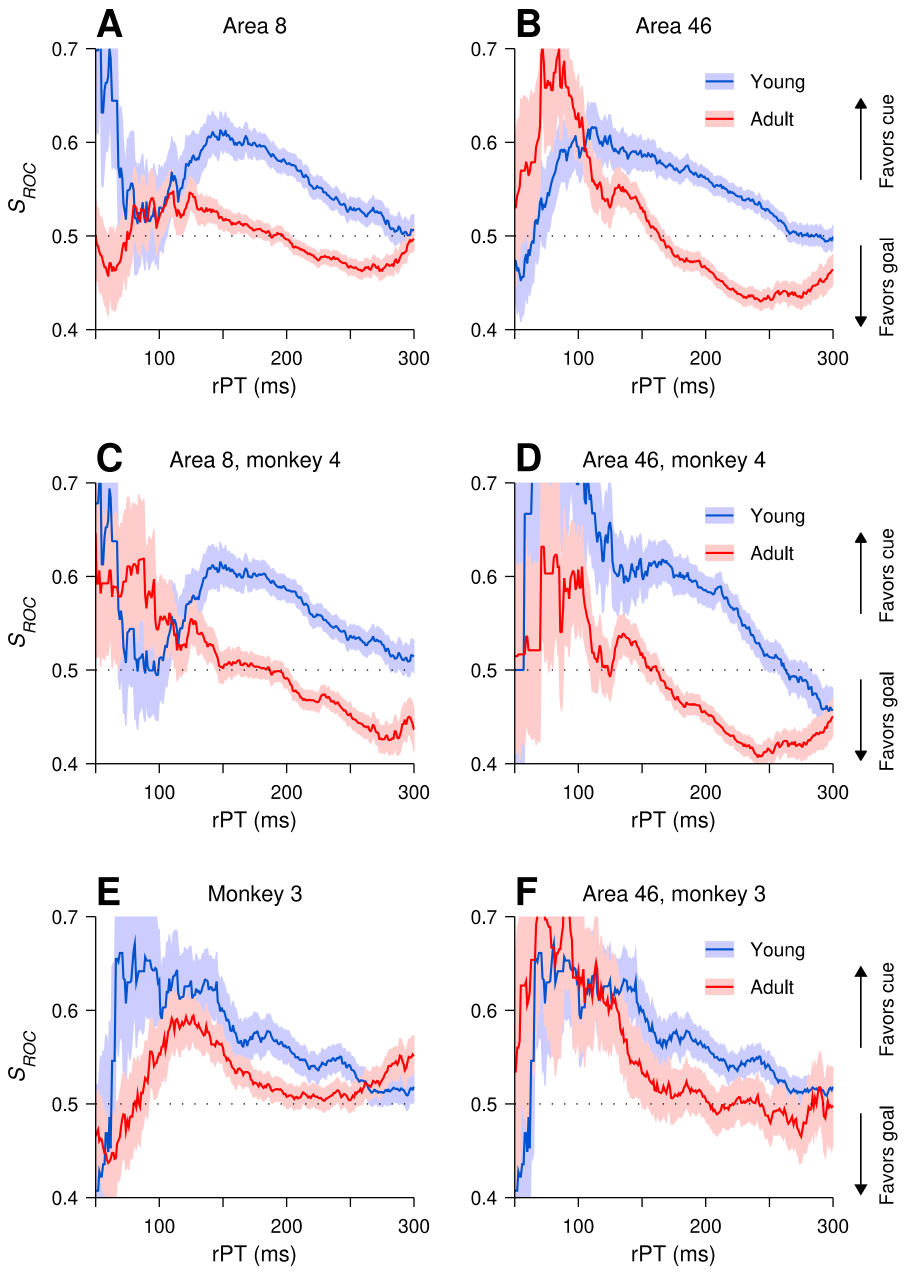

Figure S2. **Qualitatively similar age-related changes in the spatial signal were observed for prefrontal neurons recorded from areas 8a and 46, and from individual monkeys. Related to Figures 7, 8.**

(**A**, **B**) Neurometric curves based on VM neurons recorded from area 8a (A) and area 46 (B) for all monkey subjects combined. (**C**, **D**) Neurometric curves based on VM neurons recorded from area 8a (C) and area 46 (D) from monkey 4. (**E**) Neurometric curves based on all VM neurons (Areas 8a and 46) recorded from monkey 3. (**F**) Neurometric curves based on VM neurons recorded from area 46 from monkey 3. All *SROC* values were generated as described for Fig. 7E. Age-related changes were consistent across subsets of the recorded data.
